# From gut to pancreas: Shared genetic susceptibility and biological convergence in acute pancreatitis and Crohn’s disease

**DOI:** 10.1371/journal.pone.0353031

**Published:** 2026-09-11

**Authors:** Wenbin Xu, Aocheng Ji, Lu Han, Hao Xiong, Yiyi Jin, Wen Lin, Yonghui Wu, Yun Wang, Huan Zhong, Longxin Xiong, Chunyan Zeng

**Affiliations:** 1 Department of Gastroenterology, Jiangxi Province Hospital of Integrated Chinese and Western Medicine (Central Hospital of Nanchang City), Nanchang, Jiangxi, China; 2 Department of Clinical Medicine, Jiangxi University of Chinese Medicine, Nanchang, Jiangxi, China; 3 Department of Gastroenterology, Jiangxi Provincial Key Laboratory of Digestive Diseases, Jiangxi Clinical Research Center for Gastroenterology, Digestive Disease Hospital, The First Affiliated Hospital, Jiangxi Medical College, Nanchang University, Nanchang, Jiangxi, China; 4 Postdoctoral Innovation Practice Base, The First Affiliated Hospital, Jiangxi Medical College, Nanchang University, Nanchang, Jiangxi, China; 5 Eye Hospital of China Academy of Chinese Medical Sciences, Beijing, China; 6 Department of Biochemistry and Molecular Biology, University of British Columbia, Vancouver, British Columbia, Canada; 7 Department of General Surgery, Central Hospital of Nanchang City, Nanchang, Jiangxi, China; Charite Universitatsmedizin Berlin, GERMANY

## Abstract

**Background:**

Acute pancreatitis (AP) and Crohn’s disease (CD) exhibit overlapping clinical presentations and an unexpectedly high rate of comorbidity. Whether this reflects shared genetic susceptibilities remains unclear.

**Methods:**

We performed a cross-trait genome-wide association analysis leveraging European-ancestry summary statistics for AP (Ncases = 8446; Ncontrols = 437,418) and CD (Ncases = 12,194; Ncontrols = 28,072). Firstly, cross-trait genetic correlation was estimated using linkage disequilibrium score regression (LDSC) and high-definition likelihood (HDL). Secondly, to pinpoint specific pleiotropic loci and prioritize candidate genes, we employed PLACO under a rigorous composite null hypothesis, integrated with Bayesian colocalization and SMR/HEIDI analyses. Finally, we dissected the underlying biological context by mapping tissue-specific regulatory enrichment and pathway convergence using FUMA, MAGMA, and Stratified LD Score Regression (S-LDSC).

**Results:**

AP and CD showed significant positive genetic correlation (LDSC: r_g_ = 0.178, SE = 0.079, P = 0.025; HDL: r_g_ = 0.294, SE = 0.096, P = 0.0021). Pleiotropy analyses revealed 86 SNPs and 6 independent genome-wide significant pleiotropic loci (lead variants at 5q33.1, 6q22.33, 7q34, 10q24.2, 15q22.33 and 19q13.11, P_PLACO_ < 5 × 10^−8^). Colocalization showed suggestive evidence of a shared causal signal at 6q22.33 (PP4 = 0.666). Gene-based tests of the AP-CD cross-trait statistics prioritized eight pleiotropic genes—*RSPO3*, *ATG16L1*, *SMAD3*, *FADS1*, *ZPBP2*, *FADS2*, *PRKAA1* and *IRGM*. Gene-set analyses highlighted IL-23/Th17-related and broader inflammatory response pathways.

**Conclusions:**

AP and CD share polygenic susceptibility and converge on immune and inflammatory processes, with prioritized genes pointing to autophagy, lipid metabolism and TGF-β/SMAD-related biology.

## 1. Introduction

Crohn’s disease (CD), a major subtype of inflammatory bowel disease (IBD), is a chronic granulomatous enteritis involving the full thickness of the intestinal wall, often leading to strictures, fistulae and malnutrition [1,2]. Acute pancreatitis (AP), by contrast, is defined by sudden inflammatory injury to the pancreatic parenchyma that triggers the release of digestive enzymes and a cascade of local and systemic immune responses [3,4]. Despite their organ-specific pathology, CD and AP share clinical features including abdominal pain, diarrhea, weight loss and systemic inflammatory responses, and they are frequently encountered in the same patients [5]. In a Danish 16-year nationwide cohort, the standardized incidence ratio (SIR) for acute pancreatitis was 4.3 (95% CI 2.9–6.1) in Crohn’s disease, compared with the general population [6]. A meta-analysis of IBD cohorts further estimated a risk ratio of 3.62 for AP in CD [7]. These epidemiological observations raise the possibility of shared susceptibility factors beyond gallstones or medication toxicity [8].

Over the past three decades, the global prevalence of IBD has nearly doubled, reaching 6.8 million [9], while the incidence of pancreatitis has simultaneously surged to over 2.7 million cases [10]. Despite this rising burden, the biological basis of AP in patients with IBD remains incompletely clarified. Previous Mendelian randomization studies have explored the causal links between these traits [11–13]. While analyses support CD as a causal risk factor for AP, evidence regarding the reverse direction remains inconsistent [12] and these studies did not explore the specific shared genetic architecture. Notably, genetic evidence is also asymmetric: following the seminal discovery by Hugot et al. (2001) linking *NOD2* to Crohn’s disease susceptibility [14], the International IBD Genetics Consortium (IIBDGC) has cataloged over 200 risk loci [15], including *IL23R*, *JAK2* and *STAT3*, together with immune-response regions such as the HLA region and TNFSF15/TL1A [16]. More recent genetic studies have identified rare variants in inflammatory bowel disease, including variants implicating *SLC39A8* and *PLCG2* [17]. However, AP risk loci remain comparatively sparse and heterogeneous. GWAS has identified reproducible common-variant signals near *PRSS1–PRSS2* and *CLDN2*, but overall locus yield remains modest relative to CD [18]. Thus, a cross-trait framework is required to move beyond MR and single-trait AP signals by quantifying genome-wide sharing and localizing the loci and pathways that underpin AP-CD comorbidity.

**Fig 1:**
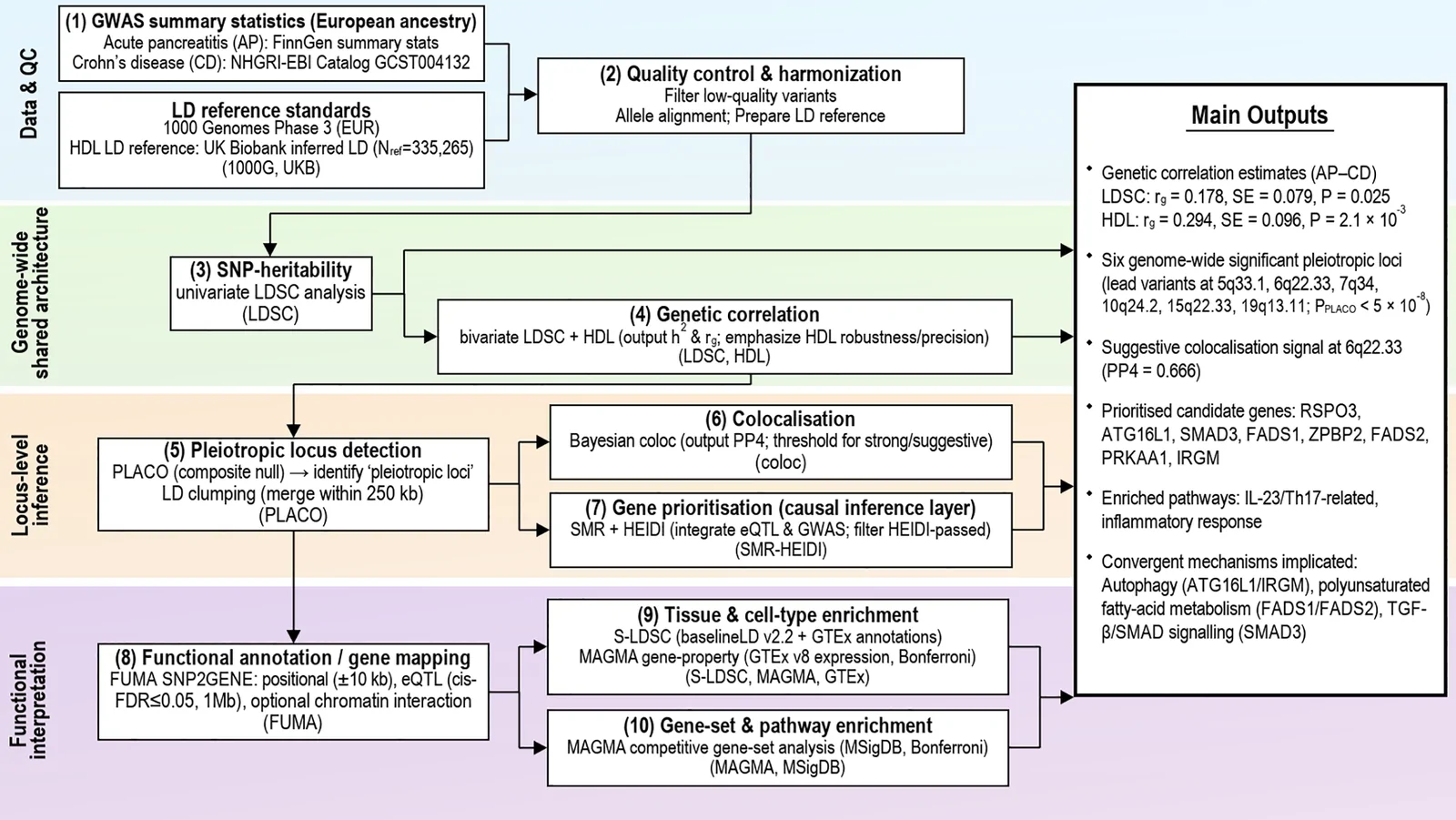
Study design and analytical workflow for cross-trait genetic analyses of acute pancreatitis and Crohn’s disease. Publicly available GWAS summary statistics of European ancestry were quality controlled and harmonized, and ancestry-matched LD reference panels were specified for downstream analyses. SNP-based heritability and genome-wide genetic correlation were estimated using LDSC and HDL. Pleiotropic loci were identified using PLACO and summarized as independent genome-wide significant regions. Locus-level inference and gene prioritization integrated Bayesian colocalization, SMR/HEIDI, and functional mapping in FUMA (positional, eQTL and chromatin-interaction mapping). Tissue/cell-type relevance and biological pathways were evaluated using S-LDSC, MAGMA gene-property and competitive gene-set enrichment analyses. Abbreviations: AP, acute pancreatitis; CD, Crohn’s disease; LDSC, linkage disequilibrium score regression; HDL, high-definition likelihood; PLACO, pleiotropy analysis under composite null hypothesis; SMR, summary-data-based Mendelian randomization; GTEx, Genotype-Tissue Expression.

Our objective was to delineate the shared genetic architecture of AP and CD and further to explore specific pleiotropic susceptibility genes by integrating biological studies and statistical genetic evidence. We first quantified genome-wide genetic correlation between AP and CD using linkage disequilibrium score regression (LDSC) and high-definition likelihood (HDL). Subsequently, Pleiotropic Analysis under Composite Null Hypothesis (PLACO) method was utilized to systematically identify pleiotropic loci between the two phenotypes, followed by locus-level Bayesian colocalization (coloc) and summary data-based Mendelian randomization (SMR) with HEIDI filtering to prioritize candidate genes supported by cis-eQTL evidence. To provide biological context, we performed functional mapping and annotation of pleiotropic loci using FUMA, conducted gene-based and gene-set analyses using MAGMA, and assessed tissue-specific enrichment using stratified LD score regression (S-LDSC) as well as MAGMA gene-property analysis based on GTEx expression profiles. Finally, we synthesized these results to propose testable mechanistic hypotheses and potential clinical implications for AP-CD comorbidity. Our study reveals shared genetic signals linking intestinal and pancreatic inflammation and provides a framework for understanding the gut–pancreas axis in complex disease. An overview of the study design and analytical workflow is provided in Fig 1.

## 2. Methods

### 2.1 Data sources and quality control

GWAS summary statistics. We analyzed publicly available GWAS summary statistics for AP and CD restricted to individuals of European ancestry. AP data were drawn from the FinnGen R12 GWAS dataset (https://r12.finngen.fi/pheno/K11_ACUTPANC), comprising 8446 cases and 437418 controls. CD summary statistics were obtained from the GWAS Catalog database (https://www.ebi.ac.uk/gwas/studies/GCST004132), with 12194 cases and 28072 controls.

Quality control. Publicly available GWAS summary statistics were obtained from their respective sources, which had implemented standard upstream genotype and imputation quality control. In our downstream processing, we additionally filtered variants to MAF ≥ 0.01 during data import and removed the extended MHC region (chr6:25–35 Mb). We harmonized effect alleles across datasets and applied method-specific filters.

For FinnGen summary statistics, genomic coordinates were harmonized to the GRCh37/hg19 build, and variants were further restricted to common variants (MAF ≥ 0.01). For LDSC, summary statistics were formatted using HapMap3 SNPs and analyzed with the 1000 Genomes Phase 3 European LD-score reference. Variants with extremely large association statistics were removed ($Z^{\text{2}}>\text{8}\text{0}$) for PLACO. For HDL, genetic correlation was estimated using the default UK Biobank imputed LD reference (Nref = 335,265). Functional annotation and gene-based analyses via FUMA/MAGMA used ancestry-matched, precomputed LD structure based on the 1000 Genomes Project reference panel.

### 2.2 Heritability and genetic correlation estimation

We first applied univariate linkage disequilibrium score regression (LDSC) [19] to estimate SNP-based heritability (h^2^) for AP and CD. LDSC regresses GWAS χ² statistics against variant-specific LD scores to partition true polygenic signal from confounding; the intercept quantifies the mean contribution of confounding bias to test-statistic inflation. We then implemented bivariate LDSC to estimate the genetic correlation (r_g_) between AP and CD. To improve precision, we applied high-definition likelihood (HDL) method [20], a full likelihood-based approach that utilizes the LD matrix and Z-score covariance; HDL uses eigen-decomposition of the LD matrix for numerical regularization and typically produces smaller standard errors than LDSC [20].

### 2.3 Identification of pleiotropic loci

We applied pleiotropic analysis under composite null hypothesis (PLACO) method [21] to identify genomic loci harboring shared genetic risk variants for both AP and CD. PLACO tests whether a SNP is associated with both traits by evaluating the product of Z-scores from two GWAS under a composite null hypothesis that the SNP affects none or only one trait. P-values are computed from a mixture of normal distributions and adjusted for correlation between summary statistics. Variants with $PPLACO\;<\;\text{5}\;\times \;{\text{1}\text{0}}^{-\text{8}}$ were considered genome-wide significant pleiotropic loci. Independent significant SNPs were defined at *r*^*2*^ *< 0.6*, lead SNPs at *r*^*2*^ *< 0.1*, and nearby LD blocks within 250 kb were merged into a single locus. Furthermore, to account for potential correlation between the summary statistics arising from shared control samples or genetic correlation, we estimated the covariance of Z-scores and applied a decorrelation procedure prior to testing. All significant loci were annotated and mapped to genes via Functional Mapping and Annotation (FUMA) [22].

### 2.4 Functional annotation and gene prioritization

Significant pleiotropic variants were annotated and mapped to genomic risk loci using the FUMA SNP2GENE pipeline. FUMA [22] first identified independent significant SNPs ($P\;<\;\text{5}\;\times \;{\text{1}\text{0}}^{-\text{8}}$) that are approximately independent at *r² < 0.6*. Genomic risk loci were then defined by merging LD blocks of independent significant SNPs located within 250 kb [23]. To prioritize candidate genes, we applied positional mapping based on physical proximity to genes (variants within 10 kb of gene boundaries) and eQTL mapping based on significant cis-eQTL associations (FDR ≤ 0.05*; up to 1 Mb*), using related tissues such as the gut, blood, and pancreas (GTEx v8) and eQTL resources[24].

### 2.5 Colocalization and SMR/HEIDI analyses

To discern whether pleiotropic loci were driven by a single shared causal variant or by distinct variants in LD, we performed Bayesian colocalization analysis [25]. For each FUMA-defined genomic risk locus, we performed colocalization within the locus boundaries reported in the GenomicRiskLoci table and used the locus lead SNP (rsID) as the index variant and computed posterior probabilities for five hypotheses: H_0_ (no association), H_1_ (AP only), H_2_ (CD only), H_3_ (both traits but distinct causal variants) and H_4_ (both traits share a causal variant). We considered PP_4_ > 0.8 as strong evidence for a shared causal variant, and PP_4_ ≥ 0.6 as suggestive evidence [26]. We further prioritized candidate genes using summary data-based Mendelian randomization (SMR) [27], which integrates summary-level statistics from GWAS with cis-expression quantitative trait loci (cis-eQTL) and tests for a pleiotropic association between gene expression and the trait using the top cis-eQTL as an instrumental variable. SMR and Heterogeneity in Dependent Instruments analysis (HEIDI) was performed using eQTLGen and six GTEx v8 tissue eQTL panels relevant to AP-CD biology, including pancreas, colon sigmoid, small intestine terminal ileum, spleen, liver, and whole blood. We applied the HEIDI heterogeneity test to identify SMR signals consistent with a single shared variant rather than linkage between distinct causal variants; results with PHEIDI > 0.05 and PSMR < 0.05 were retained. In parallel, MAGMA gene-based associations were Bonferroni corrected across all tested genes, and genes with *Pbon < 0.05* were retained for integrated prioritization.

### 2.6 Tissue and cell-type enrichment

We investigated tissue- and cell-type-related enrichment using two complementary approaches. For stratified LD score regression (S-LDSC) [28], we partitioned SNP heritability across annotations using the baseline-LD v2.2 model and the GTEx.ldcts panel, and quantified enrichment as the proportion of heritability explained by an annotation divided by the proportion of SNPs it contains; significance was assessed from the regression coefficients with multiple-testing correction across annotations. Second, we assessed tissue-specific expression enrichment using Multi-marker Analysis of GenoMic Annotation (MAGMA) [29] by regressing genome-wide gene-level association statistics on GTEx v8 tissue expression profiles; significance was assessed using a Bonferroni-corrected threshold of *P < 9.3 ×* *10*^*−4*^(0.05/54). These tissue-level enrichment and expression results are summarized in Fig 5.

### 2.7 Gene-set and pathway enrichment

We performed MAGMA competitive gene-set analysis to test whether genes in predefined biological pathways and Gene Ontology (GO) categories show stronger association signals than genes outside each set. Gene sets were drawn from MSigDB [30] collections. Statistical significance was evaluated using the competitive test framework and corrected for multiple testing across gene sets using Bonferroni adjustment; gene sets meeting the Bonferroni significance threshold of  $P\;<\text{2}.\text{9}\text{4}\;\times \;{\text{1}\text{0}}^{-\text{6}}\;(\text{0}.\text{0}\text{5}/\text{1}\text{7}\text{0}\text{0}\text{1})$ were considered significantly enriched. To further contextualize pathway-level signals, we complemented MAGMA with external database enrichment using Gene Ontology (Metascape) and GWAS Catalog gene sets (FUMA GENE2FUNC) (Fig 6). As a complementary external gene-list annotation, the eight MAGMA-prioritized AP-CD candidate genes were submitted to Metascape for Gene Ontology enrichment analysis using default significance criteria, including P < 0.01, minimum count ≥ 3, enrichment factor > 1.5, and Benjamini-Hochberg correction.

**Fig 2:**
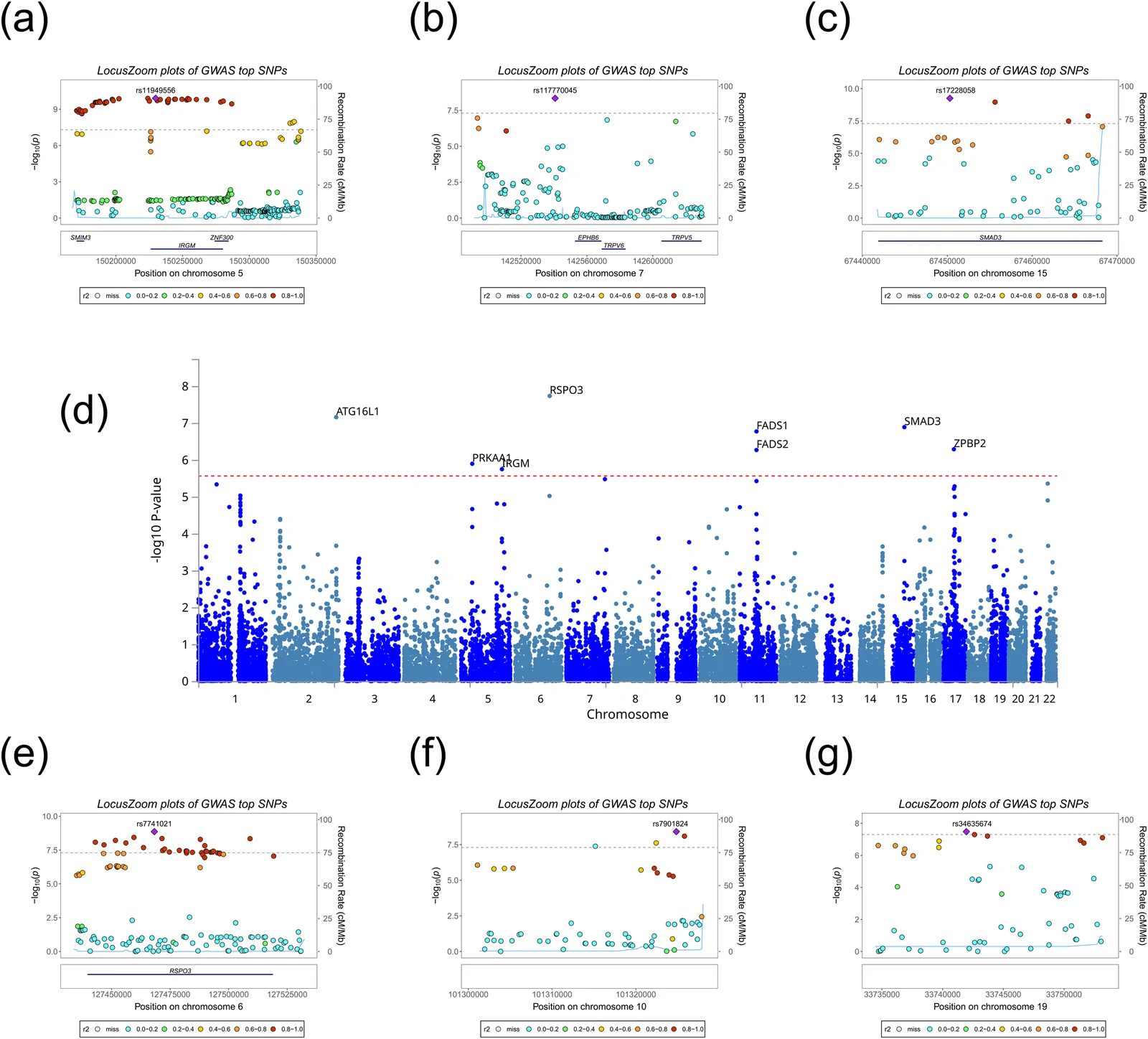
Regional association patterns of genome-wide significant pleiotropic loci and gene-level signals in the AP–CD cross-trait analysis. Panels (a–c, e–g) show LocusZoom regional plots for the six PLACO-identified pleiotropic loci, displaying the − log10(P) association signal across genomic position. In each regional plot, the lead variant is indicated at the top (rsID shown in the panel title), and surrounding variants are colored by linkage disequilibrium (r^2^) with the lead variant (color scale at the bottom). The recombination rate (cM/Mb) is plotted as a line on the right y-axis, and gene models within the locus are shown in the lower track. The horizontal dashed line denotes the conventional genome-wide significance threshold (P = 5 × 10^−8^) on the − log10(P) scale. Specifically, (a) chr5 locus indexed by rs11949556 (5q33 region; gene track includes *IRGM*), (b) chr7 locus indexed by rs117770045 (7q34 region; gene track includes *EPHB6/TRPV6/TRPV5*), (c) chr15 locus indexed by rs17228058 (15q22 region; gene track includes *SMAD3*), (e) chr6 locus indexed by rs7741021 (6q22 region; gene track includes *RSPO3*), (f) chr10 locus indexed by rs7901824 (10q24 region), and (g) chr19 locus indexed by rs34635674 (19q13.11 region). Panel (d) summarizes the cross-trait gene-based association results across the genome, where each point represents a gene and the red dashed line indicates the Bonferroni-corrected significance threshold for gene-level testing. Genes highlighted above the threshold include biologically interpretable candidates mapping to the proposed gut–pancreas axis mechanisms, notably autophagy-related genes (*ATG16L1*, *IRGM*), fibrosis/TGF-β signaling (*SMAD3*), and polyunsaturated fatty-acid metabolism (*FADS1/FADS2)*.

**Fig 3:**
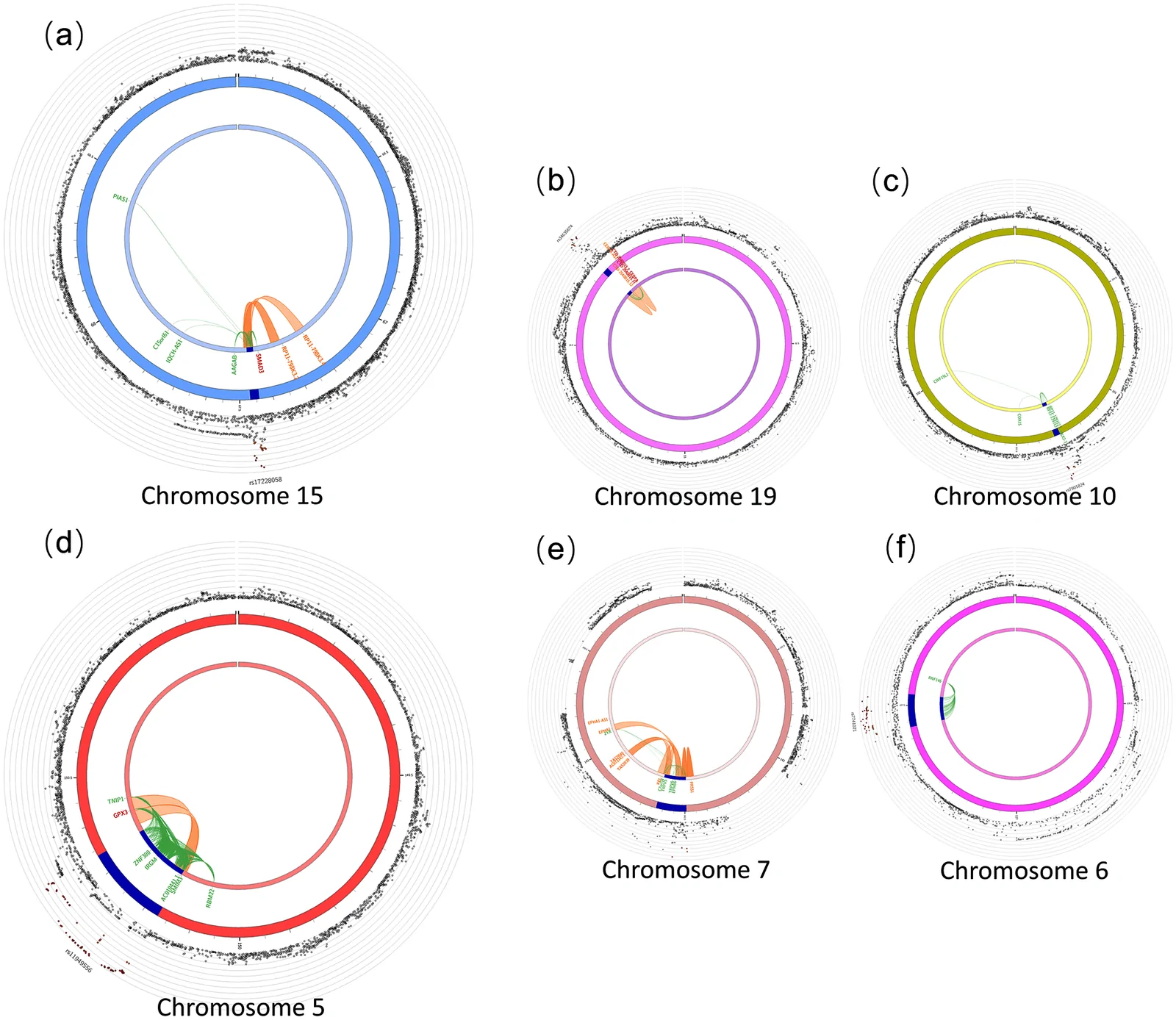
Circos visualization of regulatory mapping for six AP–CD pleiotropic loci. Circos plots summarize locus-level functional mapping for the six genome-wide significant pleiotropic loci identified by PLACO (AP–CD). For each locus, association signals across the region are displayed with linkage disequilibrium (LD) structure relative to the lead variant (color scale: pairwise r^2^), and candidate genes are shown along the genomic track. Green links denote cis-eQTL connections between associated variants and target gene expression in the selected disease-relevant tissues/cell types, whereas orange links denote significant 3D chromatin interactions connecting the locus to distal gene promoters/regulatory regions (Hi-C/chromatin interaction datasets as specified in the FUMA SNP2GENE mapping settings). (a) Chromosome 15 locus (15q22.33; lead variant rs17228058), highlighting regulatory context around SMAD3. (b) Chromosome 19 locus (19q13.11; lead variant rs34635674). (c) Chromosome 10 locus (10q24.2; lead variant rs7901824). (d) Chromosome 5 locus (5q33.1; lead variant rs11949556), highlighting regulatory context around IRGM (autophagy-related candidate). (e) Chromosome 7 locus (7q34; lead variant rs117770045). (f) Chromosome 6 locus (6q22.33; lead variant rs7741021), highlighting regulatory context around RSPO3.

**Fig 4:**
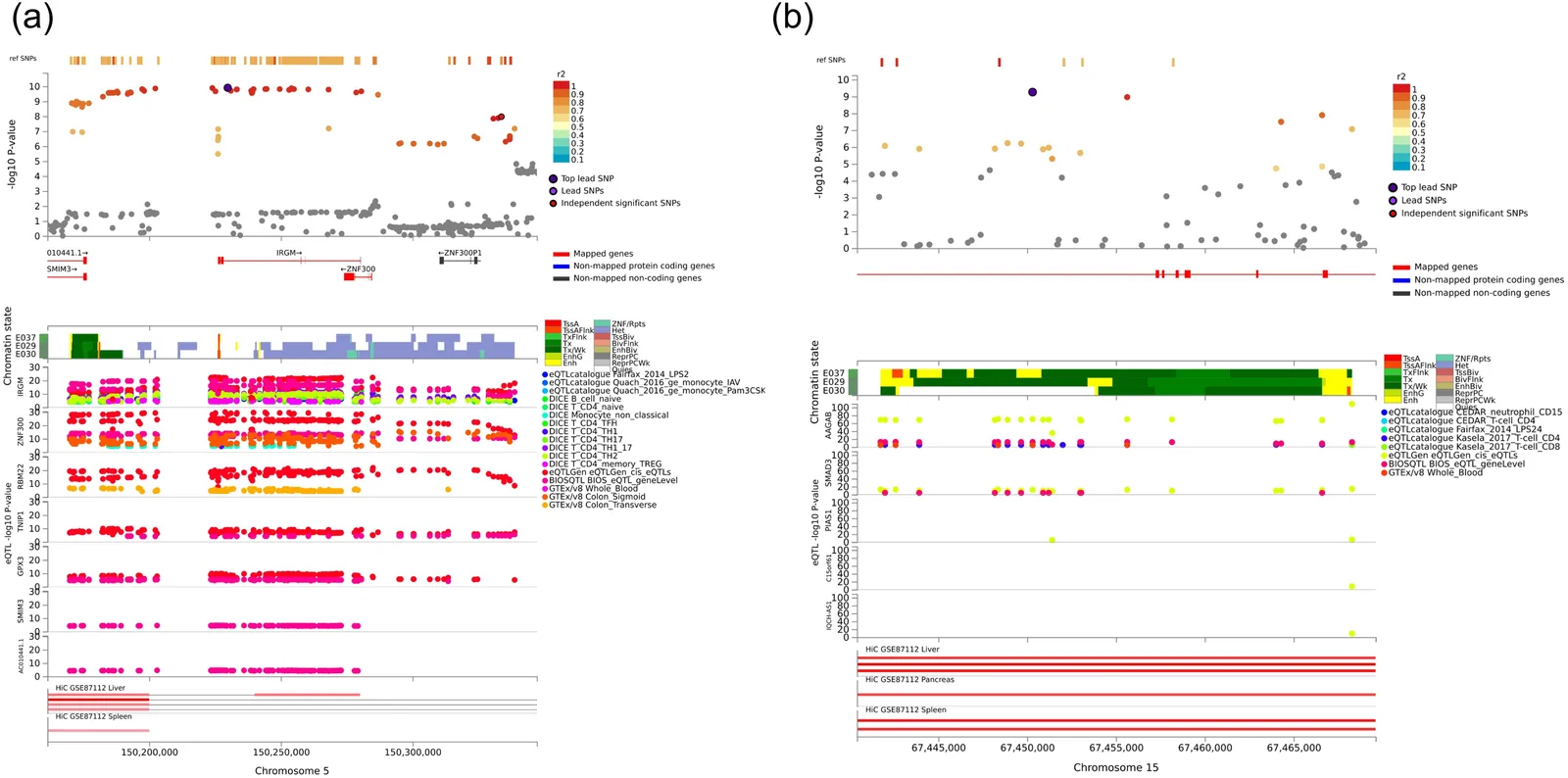
Multi-layer functional annotation of two AP-CD pleiotropic loci related to autophagy and TGF-β/SMAD signaling. (a) IRGM locus (5q33.1; lead SNP rs11949556). The top panel shows the regional association signal (−log10 P) across genomic position. Variants are colored by linkage disequilibrium (r^2^) with the lead variant, and lead or independent significant SNPs are indicated according to the plot legend. The gene track depicts local gene models, with FUMA-mapped genes highlighted. The Roadmap 15-state chromatin-state track is displayed for selected immune-relevant epigenomes, including E037 (CD4 + T helper memory cells), E029 (primary monocytes), and E030 (primary neutrophils). The eQTL track shows significant eQTL signals across immune and gastrointestinal datasets, and the bottom track shows significant Hi-C chromatin interactions (GSE87112) from liver and spleen. (b) SMAD3 locus (15q22.33; lead SNP rs17228058). The same annotation layers are shown, including the GWAS regional association pattern, gene mapping track, Roadmap chromatin states (E037/E029/E030), multi-source eQTL evidence (immune datasets; GTEx v8 whole blood), and Hi-C chromatin interactions from liver, pancreas and spleen (GSE87112). Together, these panels provide descriptive locus-level functional context for autophagy-related candidate biology at IRGM and TGF-β/SMAD-related candidate biology at SMAD3.

**Fig 5:**
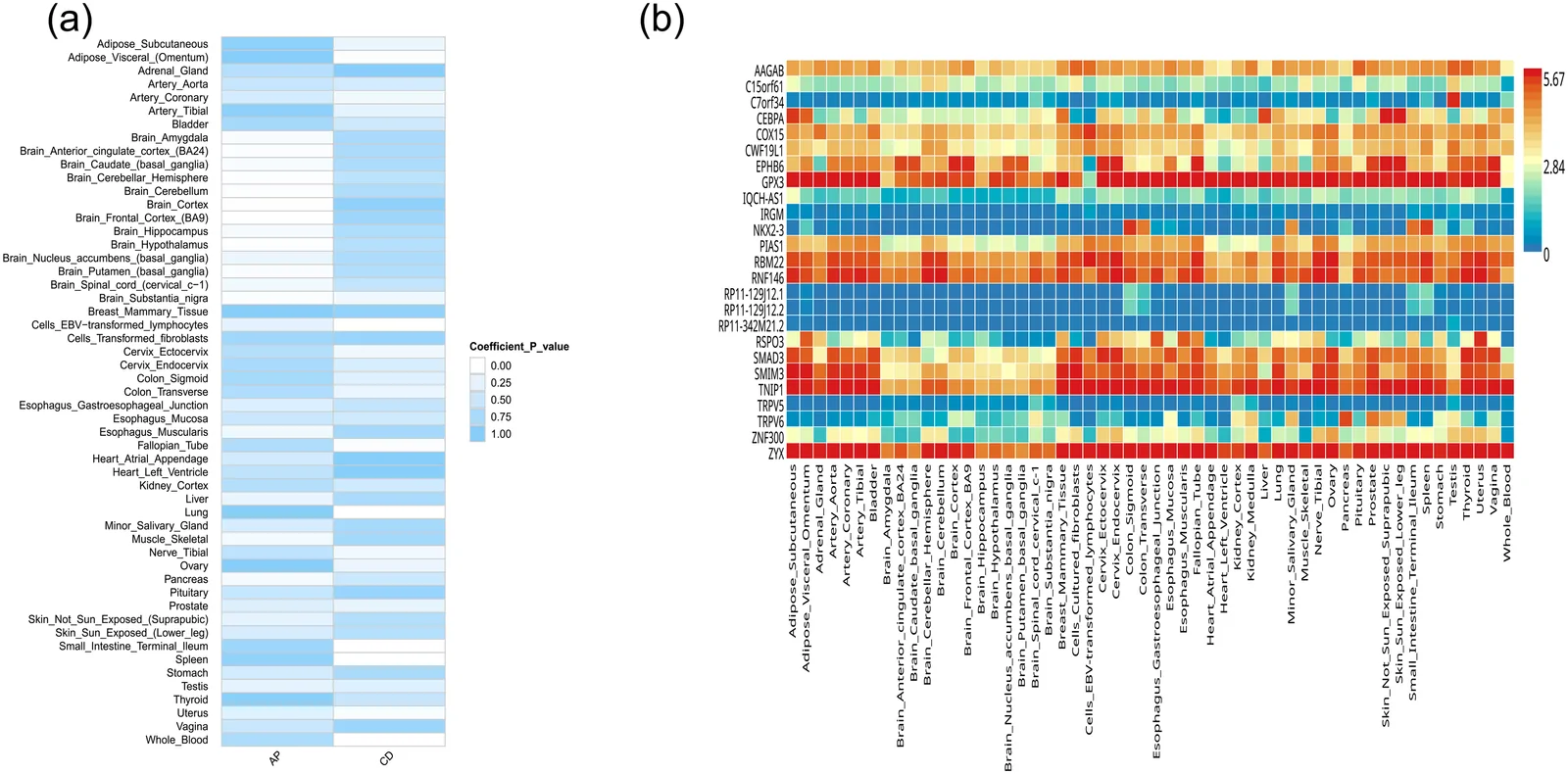
Tissue-specific heritability enrichment and gene expression profiles for acute pancreatitis (AP) and Crohn’s disease (CD). (a) Stratified LD score regression (S-LDSC) coefficient P values for AP and CD across GTEx v8 tissue annotations (white indicates smaller P values, blue indicates larger P values, as plotted). (b) GTEx v8 bulk-tissue expression heatmap for genes mapped from AP–CD pleiotropic loci using FUMA GENE2FUNC; colors represent log2-transformed expression levels scaled to the color bar. Panels provide tissue-level heritability context and descriptive GTEx expression annotation for genes mapped from AP-CD pleiotropic loci.

**Fig 6:**
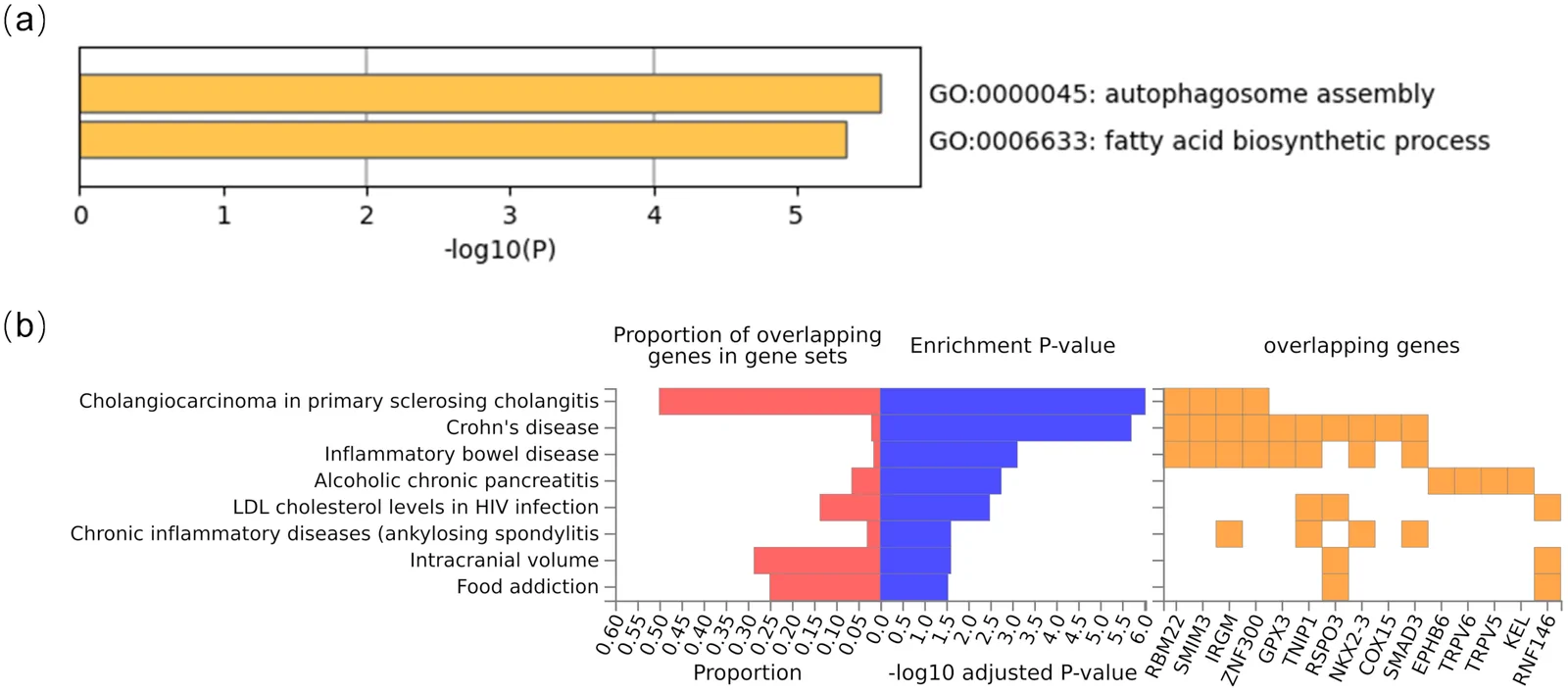
External gene-list annotation of AP-CD pleiotropy-mapped genes. (a) Gene Ontology (Biological Process) enrichment performed in Metascape using the eight MAGMA-prioritized AP-CD candidate genes. Bars indicate enrichment significance as –log10(P). The top terms include autophagosome assembly (GO:0000045) and fatty acid biosynthetic process (GO:0006633), providing gene-list-level context for autophagy- and lipid metabolism–related biological themes. (b) GWAS Catalog gene-set enrichment generated by FUMA GENE2FUNC using FUMA-mapped genes from AP-CD pleiotropic loci. The left panel shows the proportion of overlapping genes between the input list and each GWAS Catalog trait gene set; the middle panel shows the –log10 adjusted P-value for enrichment; and the right panel displays the identity of overlapping genes contributing to each enrichment signal.

## 3. Results

### 3.1 Heritability and genetic correlation between acute pancreatitis and Crohn’s disease

Bivariate LDSC yielded a moderate positive genetic correlation between AP and CD (rg = 0.178, SE = 0.079, P = 0.025) (Table 1 and S1 and S2 Tables in S1 File). HDL provided a complementary estimate, yielding rg = 0.294 (SE = 0.096, P = 0.0021) for AP-CD. These findings suggest a modest yet significant shared polygenic architecture between AP and CD.

**Table 1:** Genome-wide genetic correlation between acute pancreatitis (AP) and Crohn’s disease (CD) assessed using LDSC and HDL.

	LDSC			HDL		
Trait pairs	Rg	SE	P	Rg	SE	P
AP & CD	0.178	0.079	0.025	0.294	0.096	0.0021

Genetic correlations (Rg), standard errors (SE), and p-values (P) were computed through linkage disequilibrium score regression (LDSC) and high-definition likelihood (HDL). Both methods show statistically significant correlations (*P < 0.05*) between AP and CD. AP, acute pancreatitis; CD, Crohn’s disease.

### 3.2 Genome-wide pleiotropic loci

PLACO identified six independent genome-wide significant loci harboring SNPs associated with both AP and CD ($PPLACO\;<\;\text{5}\;\times \;{\text{1}\text{0}}^{-\text{8}}$; Fig 2 and Table 2). These loci were located on chromosomes 5q33.1 (lead SNP rs11949556), 6q22.33 (rs7741021), 7q34 (rs117770045), 10q24.2 (rs7901824), 15q22.33 (rs17228058) and 19q13.11 (rs34635674). Notably, several loci overlapped biologically plausible genes prioritized by downstream integration, including *RSPO3* at 6q22.33, *IRGM* at 5q33.1 and *SMAD3* at 15q22.33 (S7 Table in S1 File). In addition, gene-based analysis highlighted the *FADS1/FADS2* cluster and *TMEM258* at 11q12.2. Regional association plots for each pleiotropic locus, together with the cross-trait gene-based association landscape, are shown in Fig 2.

**Table 2:** Genome-wide significant AP-CD pleiotropic loci identified by PLACO and annotated by FUMA.

CHR	rsID	pos	p	start	end	nSNPs	LeadSNPs
5	rs11949556	150229801	1.24932980419e-10	150169843	150338714	278	rs11949556
6	rs7741021	127468274	1.37994684146e-09	127435106	127532807	103	rs7741021
7	rs117770045	142540716	4.54192626259e-09	142493576	142629782	5	rs117770045
10	rs7901824	101324823	3.89684200549e-09	101301093	101327851	12	rs7901824
15	rs17228058	67450305	5.51718840286e-10	67441750	67468285	22	rs17228058
19	rs34635674	33741951	3.29450065944e-08	33734695	33753166	14	rs34635674

Genomic regions showing significant pleiotropy were delineated based on PLACO cross-trait summary statistics. For each locus, the chromosome (CHR), lead SNP (rsID), genomic position (Pos), joint association P-value (p), locus boundaries (start–end), and the number of FUMA candidate SNPs assigned to the locus (nSNPs) are reported. Six independent loci were identified across chromosomes 5, 6, 7, 10, 15, and 19, all satisfying the genome-wide significance threshold ($P\;<\;\text{5}\;\times \;{\text{1}\text{0}}^{-\text{8}}$).

### 3.3 Colocalization analysis

Bayesian colocalization analysis assessed whether the AP and CD signals at each pleiotropic locus were driven by the same causal variant. The 6q22.33 locus showed suggestive evidence of a shared causal variant with posterior probability PP_4_ = 0.666. In contrast, the other loci had lower PP_4_ values (0.04–0.18), indicating that AP and CD associations in these regions may arise from distinct variants in LD or trait-specific association signals. For example, at the 5q33.1 locus the cross-trait association may be driven by different variants affecting *IRGM/TNIP1* in CD and another gene or regulatory element in AP. These results indicate that most pleiotropic regions did not show strong colocalization evidence and may reflect overlapping yet distinct genetic signals (S6 Table in S1 File).

### 3.4 Prioritized genes

Integrating cross-trait gene-based association evidence and locus-based functional mapping, we prioritized eight candidate genes potentially underlying the shared AP-CD genetic architecture: *RSPO3, ATG16L1, SMAD3, FADS1, ZPBP2, FADS2, PRKAA1* and *IRGM* (Table 3 and S8 Table in S1 File). All eight reached Bonferroni significance in the AP-CD cross-trait gene-based test. SMR/HEIDI further supported a subset of these AP-CD prioritized candidates, including *IRGM* in eQTLGen, *FADS1* in GTEx pancreas and colon sigmoid, and *FADS2* in GTEx pancreas. Several are established immune-mediated disease genes and pathways, including autophagy-related CD genes (*ATG16L1* [31] and *IRGM* [32]), lipid desaturation genes central to PUFA and eicosanoid biosynthesis (*FADS1/FADS2* [33]), and the TGF-β effector *SMAD3* [34] implicated in fibrogenic responses. *PRKAA1* [35] encodes the *AMPK* [36] catalytic α1 subunit, a key immunometabolic regulator. *RSPO3* [37] is an LGR ligand that potentiates canonical Wnt/β-catenin signaling and is a known driver of mucosal healing and epithelial regeneration. *ZPBP2* [38] lies within the 17q21 regulatory block near *ORMDL3/GSDMB*, a locus repeatedly implicated in immune-related traits. To visualize how pleiotropic signals at each locus may link to specific genes, we summarized locus-level regulatory evidence by integrating cis-eQTL links and 3D chromatin interactions in circos plots for all six genome-wide pleiotropic loci (Fig 3). To provide locus-level functional context for two mechanistically informative pleiotropic signals, we subsequently visualized multi-layer regulatory annotations at the IRGM (5q33.1) and SMAD3 (15q22.33) loci using FUMA regional plots integrating GWAS association, Roadmap chromatin states, eQTL evidence, and Hi-C chromatin interactions (Fig 4).

**Table 3:** Bonferroni-significant MAGMA gene-based candidates and SMR/HEIDI support across eQTL panels.

Gene	Ensembl ID	Chr	Position (hg19)	*P*_*MAGMA*_	*P*_*bon*_	eQTLGen	GTEx Pancreas	GTEx Colon sigmoid
ATG16L1	ENSG00000085978	2	234,118,697−234,204,320	6.97E-08	1.27E-03	0	0	0
FADS1	ENSG00000149485	11	61,567,099−61,596,790	1.69E-07	3.08E-03	0	1	1
FADS2	ENSG00000134824	11	61,560,452−61,634,826	5.40E-07	9.86E-03	0	1	0
IRGM	ENSG00000237693	5	150,226,085-150,280,298	1.79E-06	3.26E-02	1	0	0
PRKAA1	ENSG00000132356	5	40,759,481−40,798,476	1.27E-06	2.32E-02	0	0	0
RSPO3	ENSG00000146374	6	127,439,749−127,518,910	1.84E-08	3.36E-04	0	0	0
SMAD3	ENSG00000166949	15	67,356,101−67,487,533	1.29E-07	2.36E-03	0	0	0
ZPBP2	ENSG00000186075	17	38,024,417−38,034,149	5.14E-07	9.39E-03	0	0	0

Genes were included based on MAGMA gene-based Bonferroni significance. P_MAGMA_ denotes the MAGMA gene-based P value, and P_bon_ denotes the Bonferroni-corrected MAGMA gene-based P value across all tested genes. SMR/HEIDI support is shown as binary indicators; “1” indicates that the corresponding gene–eQTL panel pair met the prespecified SMR/HEIDI criteria, whereas “0” indicates that the criteria were not met. For readability, only eQTL panels with at least one supported association are displayed in the main table.

### 3.5 Tissue-specific heritability and gene expression enrichment

Stratified LDSC demonstrated no tissue achieving FDR-significant enrichment for AP. Nominal enrichment was observed in several brain regions (frontal cortex, hypothalamus and cerebellum), but these signals did not survive multiple testing. In contrast, CD heritability was significantly enriched (FDR < 0.05) in whole blood, spleen, EBV-transformed lymphocytes, lung and terminal ileum-tissues central to systemic and mucosal immunity (Fig 5a and S3 Table in S1 File). To provide descriptive biological context for genes mapped from AP-CD pleiotropic loci, we visualized GTEx v8 bulk-tissue expression profiles generated by FUMA GENE2FUNC for annotation (Fig 5b).

### 3.6 Pathway enrichment

MAGMA competitive gene-set analysis of AP-CD cross-trait gene-level statistics identified significant enrichment in immune and inflammatory programs, including defense response, inflammatory response, inflammatory bowel disease signaling, and type-17/cytokine-mediated immune regulation (S4 and S5 Tables in S1 File). External ontology annotation of the eight prioritized AP-CD genes using Metascape highlighted two leading GO biological processes: autophagosome assembly (GO:0000045; driven by *PRKAA1*, *ATG16L1* and *IRGM*; q ≈ 0.034) and fatty acid biosynthetic process (GO:0006633; driven by *FADS1*, *FADS2* and *PRKAA1*; q ≈ 0.034) (Fig 6a).

## 4. Discussion

Our cross-trait analysis provides genetic support for a gut–pancreas axis in which CD liability and AP injury share measurable polygenic components. Beyond a modest but significant genome-wide correlation, the AP-CD overlap resolves into six independent pleiotropic loci and a small set of biologically interpretable candidate genes. Specifically, we identified six independent pleiotropic loci at 5q33.1, 6q22.33, 7q34, 10q24.2, 15q22.33 and 19q13.11 and prioritized eight candidate genes, namely *ATG16L1, IRGM, FADS1, FADS2, SMAD3, PRKAA1, RSPO3* and *ZPBP2*. However, functional support varied across genes. SMR provided regulatory evidence for *IRGM* in eQTLGen, for *FADS1* in GTEx pancreas and colon sigmoid, and for *FADS2* in GTEx pancreas, whereas colocalization at 6q22.33 offered only moderate support for a shared causal variant, falling short of a stringent colocalization threshold. Tissue-level analyses provided more limited evidence than pathway-level analyses. S-LDSC did not reveal FDR-significant tissue enrichment for AP, whereas CD showed enrichment in immune- and gut-related tissues. Cross-trait MAGMA gene-property analysis suggested a terminal-ileum expression context, and gene-set analyses implicated inflammatory and type-17 immune programs, including IL-23 signaling and Th17 lineage commitment. Together, these findings suggest that the AP-CD genetic overlap may involve immune and inflammatory biology, but this interpretation remains primarily gene- and pathway-based rather than supported by significant AP tissue-enrichment evidence.

Comparison with large IBD GWAS indicates that three of our pleiotropic loci—5q33.1 (*IRGM*), 10q24.2 (*NKX2–3*) and 15q22.33 (*SMAD3*) [39]—fall within regions repeatedly associated with CD susceptibility, and are here further implicated as shared loci between AP and CD. At the same time, three additional loci highlight routes to AP-CD comorbidity that have been less emphasized in prior genetics: 6q22.33, 7q34 and 19q13.11. The 6q22.33 locus, which showed suggestive colocalization evidence, nominates RSPO3 as a candidate gene, and multiple injury models show that vascular sources of *RSPO3* are required for efficient intestinal epithelial regeneration after damage, via Wnt/β-catenin pathway activation [40]. One testable hypothesis is that genetic variation at 6q22.33 may influence RSPO3-related injury-repair programs, although this requires experimental validation in relevant gut and pancreatic inflammatory models. At 7q34, the interval includes *TRPV6*, an epithelial calcium channel in which rare damaging coding variants have been associated with chronic pancreatitis susceptibility in prior sequencing studies [41]. It is relevant to our AP-CD signal since disordered intracellular calcium handling is an early and central feature of pancreatic acinar injury [42]; however, fine-mapping should explicitly test whether our shared signal is best explained by *TRPV6* regulation versus neighboring candidates [43], whereas the 19q13.11 signal lies near *CEBPA*, raising the possibility of myeloid involvement [44] that has been less emphasized in prior AP or CD GWAS. Collectively, these findings refine the AP-CD overlap into a combination of established CD susceptibility regions and additional candidate loci that warrant targeted fine-mapping and functional follow-up.

At the gene level, the *FADS1/FADS2* genes drew our attention. *FADS1* and *FADS2* encode the key desaturases shaping long-chain PUFA biosynthesis, and studies have shown that common variation across this cluster markedly influences ω-3/ω-6 PUFA composition [45], thereby constraining substrate availability for downstream lipid mediator production during inflammation. Considering the context of comorbidities, we propose that: if genetically determined PUFA profiles bias mediator output toward stronger or more persistent inflammatory signaling, the clinical threshold for AP may be lowered during CD-associated systemic inflammation, particularly in cytokine-high flare states. Interestingly, impaired intracellular clearance mechanisms also disrupt the intestinal barrier and exacerbate systemic innate inflammation [46], which is consistent with the functions of the prioritized genes in our results, such as *ATG16L1* and *IRGM*; *ATG16L1* is a canonical CD susceptibility gene, with functional evidence linking *ATG16L1* deficiency to abnormal Paneth-cell granule exocytosis and impaired antimicrobial peptide delivery in ileal epithelium, resulting in mucosal barrier fragility [31]. In pancreatitis models, deletion of the *ATG16L1* WD40 domain abolishes LAP-like non-canonical autophagy, slows intracellular trypsin degradation, and exacerbates experimental acute pancreatitis, implicating impaired clearance efficiency as a determinant of pancreatic vulnerability under systemic inflammatory stress [47]. Furthermore, Mehto et al. found that *IRGM* can restrain *NLRP3* inflammasome activation by hindering inflammasome assembly and promoting selective autophagic degradation of inflammasome components, thereby limiting IL-1β maturation [32]. Consistent with this axis, Metascape annotation of the eight prioritized genes recovered autophagosome assembly (GO:0000045) and fatty acid biosynthetic process (GO:0006633) among the top terms, providing a complementary, gene-list–level line of support for convergence on autophagy-linked clearance and lipid mediator biology. Together, these observations suggest a plausible hypothesis in which autophagy-linked clearance, inflammasome regulation, and lipid mediator biology may contribute to heterogeneity in pancreatic injury risk during CD-associated inflammatory states.

Notably, compared with other pleiotropic loci, colocalization support was strongest—although still moderate—at 6q22.33, making *RSPO3* a particularly tractable candidate for functional follow-up. *RSPO3* is a well-established Wnt potentiator that is induced after injury, and both stromal and lymphatic/endothelial sources of *RSPO3* have been shown to support crypt repair and epithelial regeneration in injury models [48]. Genetic variation at 6q22.33 may influence *RSPO3*-related injury-repair signaling, providing a plausible link between mucosal restitution after CD-related damage and pancreatic inflammatory susceptibility. In parallel, *SMAD3*, a core effector of TGF-β signaling, aligns with fibrogenic remodeling, and a clinical genetic study has associated *SMAD3* variation with increased risk of repeat operation and shorter time to re-operation in CD [49], consistent with a more aggressive fibrostenotic course. Collectively, these results suggest that AP-CD comorbidity may be shaped by lipid-related inflammatory processes, autophagy-linked resilience, and repair–remodeling, and they nominate intermediate phenotypes for gut–pancreas risk stratification.

Our tissue and pathway signals add a clearer context to these gene-level interpretations. In intestinal inflammation, macrophages are repeatedly identified as prominent sources of IL-23 in the lamina propria, and IL-23 is tightly linked to pathogenic Th17-like T-cell states [50]. On the pancreatic side, multiple experimental and clinical studies implicate IL-17A as an amplifier of pancreatitis severity by acting on acinar and stromal cells and promoting recruitment of innate effector cells [51]. In our study, enrichment analysis focused on the intestinal mucosa and immune-related tissues, combined with functional interpretation of pleiotropic genes, suggests that when the intestinal mucosal barrier is disrupted and type 17 inflammation is engaged, genetically driven differences in autophagy control, lipid mediator production, and repair signaling may increase the likelihood that systemic inflammation extends to clinically meaningful pancreatic injury. The most informative next steps are to resolve cell-state specificity in inflamed mucosa and myeloid compartments and to test whether the top loci RSPO3 at 6q22.33, and the 17q12-q21 regulatory block show inflammation-dependent regulatory effects that align with AP-relevant inflammatory readouts.

Clinical studies provide complementary context for the potential gut–pancreas inflammatory link. A prospective cohort study reported early derangement of gut-barrier function in severe acute pancreatitis (SAP), including increased intestinal permeability and systemic endotoxin exposure [52]. Strain-level comparisons further suggested that most pancreatic and extra-pancreatic organ infections originate in the intestine [53]. Conversely, in CD, idiopathic acute pancreatitis has been associated with higher biologic utilization and increased surgery rates [54]. Nevertheless, the directionality and causal ordering of gut–pancreas interactions remain to be resolved in dedicated longitudinal studies. From a therapeutic perspective, compared with total parenteral nutrition, initiating jejunal enteral nutrition of SAP has been reported to reduce infectious complications and mortality [55], indirectly supporting that preserving gut-barrier integrity may mitigate the clinical course of pancreatitis. Evidence for lipid-lowering interventions is more heterogeneous. Current expert recommendations for hypertriglyceridemic AP emphasize rapid triglyceride reduction in the acute phase [56]. For CD itself, although human genetics suggests that increased *LDLR* and *LPL* activity is associated with a lower risk of IBD [57], interventional trials have not yet demonstrated that lipid-lowering therapy improves CD prognosis. In routine practice, among CD patients with concomitant dyslipidemia, optimized lipid control is therefore more likely to translate into a lower risk of recurrent pancreatitis, while any benefit on intestinal symptoms remains to be established.

## 5. Limitations

Several limitations warrant consideration. First, our analyses were restricted to European-ancestry GWAS summary statistics, and replication in independent multi-ancestry cohorts is needed. Second, phenotype heterogeneity may affect locus-level specificity: AP diagnoses were not uniformly subtyped by etiology or severity, and CD definitions varied across contributing studies. Third, summary-statistics analyses cannot establish causality or directionality, and apparent pleiotropy may reflect shared biology or distinct causal variants in linkage disequilibrium. Fourth, functional interpretation depends on bulk-tissue regulatory resources largely generated under baseline conditions, so cell-state- and stimulus-dependent effects in intestinal or pancreatic compartments may be missed. Finally, we did not model gene–environment or clinical modifiers that are highly relevant to AP risk. Future work should combine larger multi-ancestry datasets, etiologic AP subtyping, context-specific QTL resources, and experimental validation in gut–pancreas models.

## 6. Conclusion

We delineate a shared AP-CD genetic architecture supported by genome-wide genetic correlation and pleiotropic association signals. Integrating pleiotropic loci with gene-mapping prioritization, we nominate a focused set of candidate genes and pathways underlying comorbidity. Lipid metabolism, intracellular clearance, and tissue-remodeling pathways emerged as candidate biological themes, but their mechanistic roles require further validation. These findings provide biological context for AP-CD comorbidity, refine the interpretation of shared genetic susceptibility, and offer a framework for future research.

## Supporting information

S1 File Comprehensive summary of AP-CD cross-trait supplementary results. This Excel file provides the complete supplementary results for the AP-CD cross-trait analyses, organized into eight worksheets, each corresponding to the relevant analyses and in-text references.
(XLSX)
